# TIMP-1 and responsiveness to gemcitabine in advanced breast cancer; results from a randomized phase III trial from the Danish breast cancer cooperative group

**DOI:** 10.1186/1471-2407-14-360

**Published:** 2014-05-22

**Authors:** Charlotte Levin Tykjær Jørgensen, Christina Bjerre, Bent Ejlertsen, Karsten D Bjerre, Eva Balslev, Annette Bartels, Nils Brünner, Dorte L Nielsen

**Affiliations:** 1 Department of Pathology, Herlev University Hospital, Herlev Ringvej 75, Herlev 2730, Denmark; 2 Sino-Danish Breast Cancer Research Centre, Section for Molecular Disease Biology, Institute of Veterinary Disease Biology, Faculty of Health and Medical Sciences, University of Copenhagen, Strandboulevarden 49, Copenhagen Ø 2100, Denmark; 3 Danish Breast Cancer Cooperative Group, Strandboulevarden 49, Copenhagen 2100 Ø, Denmark; 4 Department of Oncology, Copenhagen University Hospital, Rigshospitalet, Blegdamsvej 9, Copenhagen 2100 Ø, Denmark; 5 Department of Oncology, Herlev University Hospital, Herlev Ringvej 75, Herlev 2730, Denmark

**Keywords:** TIMP-1, Gemcitabine, Docetaxel, Breast cancer, Prediction, Prognosis

## Abstract

**Background:**

Tissue inhibitor of metalloproteinases-1 (TIMP-1) has anti-apoptotic functions, which may protect TIMP-1 positive cancer cells from the effects of chemotherapy such as docetaxel and gemcitabine. The purpose of the present study was to evaluate TIMP-1 immunoreactivity as a prognostic and predictive marker in advanced breast cancer patients receiving docetaxel (D) or gemcitabine plus docetaxel (GD).

**Methods:**

Patients with locally advanced or metastatic breast cancer who were assigned to D or GD by participation in a randomized phase III trial were included in the study. Assessment of TIMP-1 status was performed retrospectively on primary tumor whole-tissue sections by immunohistochemistry and tumor samples were considered positive if epithelial breast cancer cells were stained by the anti-TIMP-1 monoclonal antibody VT7. Time to progression (TTP) was the primary endpoint. Overall survival (OS) and response rate (RR) were secondary endpoints. Associations between TIMP-1 status and outcome after chemotherapy were analyzed by Kaplan-Meier estimates and Cox proportional hazards regression models.

**Results:**

TIMP-1 status was available from 264 of 337 patients and 210 (80%) of the tumors were classified as cancer cell TIMP-1 positive. No significant difference for TTP between TIMP-1 positive versus TIMP-1 negative patients was observed in multivariate analysis, and RR did not differ according to TIMP-1 status. However, patients with TIMP-1 positive tumors had a significant reduction in OS events (hazard ratio = 0.71, 95% confidence interval (CI) = 0.52-0.98, P = 0.03). Additionally, a borderline significant interaction for OS was observed between TIMP-1 status and benefit from GD compared to D (P_interaction_ = 0.06) such that median OS increased by nine months for TIMP-1 negative patients receiving GD.

**Conclusions:**

TIMP-1 status was an independent prognostic factor for OS but not TTP in patients with advanced breast cancer receiving either D or GD. There was no statistically significant interaction between TIMP-1 status and treatment, but a trend towards an incremental OS from the addition of gemcitabine to docetaxel in patients with TIMP-1 negative tumors suggests further investigation.

## Background

In advanced breast cancer, chemotherapy is used for patients with estrogen receptor (ER) negative, endocrine resistant, or rapidly progressive disease to offer symptom control and improve survival. Whether to use combination chemotherapy or a sequential single agent chemotherapy strategy remains unclear. Combination chemotherapy is associated with higher response rates (RR) and improved time to progression (TTP) but the survival benefit is at its best modest and often linked with increased toxicity [1]. Therefore there is a need for tools that can identify those patients who will benefit the most from combination chemotherapy.

Breast cancer is recognized as a heterogeneous disease and response to treatment seems to depend on molecular characteristics of the tumor, some of which confer resistance to specific drugs while others confer a more multiresistant phenotype covering several different drug classes [2-8]. Predictive markers may serve as tools for tailoring therapy for individual patients, yet the number of clinically useful markers is still limited [9-11].

Tissue inhibitor of metalloproteinases-1 (TIMP-1) is a multifunctional protein, where some of its functions are related to the inhibition of matrix metalloproteinases (MMPs) while other biological functions are MMP-independent, such as inhibition of apoptosis and stimulation of proliferation [12-16]. A prognostic value of TIMP- 1 in primary breast cancer has been suggested in several studies, with high plasma or tumor tissue content of TIMP-1 being associated with poor patient outcome [15-19]. Moreover, breast cancer patients with TIMP-1 positive cancer cells [2,8,20,21] seem to benefit less from adjuvant anthracycline-containing chemotherapy. Docetaxel (D), a taxane disrupting the dynamic function of microtubules [22], and gemcitabine (G), a pyrimidine analog arresting DNA replication and synthesis [23-25], are widely used in breast cancer therapy [26,27]. A phase III clinical trial by the Danish Breast Cancer Cooperative Group (DBCG) [28] compared the efficacy of D versus GD in patients with locally advanced or metastatic breast cancer. GD increased TTP by two months compared to D alone, while RR and overall survival (OS) were similar [28]. The purpose of the present study was to assess the potential predictive and prognostic information provided by TIMP-1 in patients participating in this trial. We have previously shown a differential benefit in these patients from the addition of G to D depending on intrinsic molecular subtype [29], and consequently we additionally sought to clarify whether a possible effect of TIMP-1 was independent of intrinsic subtypes.

## Methods

### Patients

The present study was based upon a DBCG randomized, phase III, multicenter trial previously described in detail [28]. The trial compared the efficacy of D to the combination of GD in 337 patients with histologically confirmed locally advanced or metastatic breast cancer. Patients were randomly assigned to D (100 mg/m^2^) day 1, every 21 days, or G (1000 mg/m^2^) days 1 and 8 plus D (75 mg/m^2^) day 8, every 21 days. Patients were either previously untreated, had received prior (neo)adjuvant chemotherapy or a single prior chemotherapy regimen, mostly anthracycline-based, for metastatic breast cancer. The majority of patients had *HER2* normal (68.8%) and hormone receptor positive disease (70.9%). More than half of the patients had visceral disease (57.3%). The type and amount of post-study chemotherapy were similar in the two arms. The study was conducted in accordance with the Declaration of Helsinki, and all patients gave their signed informed consent prior to study entry. DBCG prepared the original protocol as well as the biomarker supplement, and the Danish National Committee on Biomedical Research Ethics approved the original protocol and the supplement (KF 02-045-01 and KF 12 315632/H-KF-02-045-01) prior to activation.

### TIMP-1 immunohistochemical staining

Expression of TIMP-1 protein was evaluated on formalin-fixed, paraffin-embedded (FFPE) primary tumor tissue whole sections (3 μm). The validated mouse monoclonal antibody (clone VT7) raised against recombinant human TIMP-1 [30,31] was applied for immunohistochemical (IHC) staining as previously described [8]. In brief, sections were deparaffinized in xylene and rehydrated in graded concentrations of ethanol. For antigen retrieval, the sections were microwave treated in citrate buffer pH = 6 and endogen peroidase activity was blocked by hydrogen peroxide. Sections were incubated with VT7 (0.25 ug/ml) overnight at 4°C, and the antibody was detected with mouse/rabbit Advanced HRP (Code No. 4068, Dako A/S), and the reaction was visualized with DAB + (Code No. K5007, Dako A/S). TIMP-1 was assessed semi quantitatively using the positive (any cytoplasmatic staining of tumor cells, > 0%) versus negative (no staining of tumor cells) staining signal as a measure of the TIMP-1 immunoreactivity in the epithelial breast cancer cells [2,8,20]. The whole-tissue sections were scanned and examined by light microscopy and reviewed blinded, without knowledge of patient characteristics and outcome, by three independent investigators (pathologist EB and two trained observers, technician AB and biologist CLTJ). The independent scores from all three investigators were consolidated into a final score. In case of discrepancies, agreement was reached by the three investigators evaluating the slides together.

### Statistics

Associations between TIMP-1 protein status and prognostic and demographic variables of the main study [28] and PAM50 intrinsic subtype [29] were assessed. Associations between TIMP-1 and categorical variables (treatment regimen, hormone receptor status, human epidermal growth factor receptor 2 (*HER2*) status, type of metastatic site, stage of disease, previous chemo-, hormonal-, and radiation-therapy, and PAM50 intrinsic subtype) were evaluated by Fisher’s exact test, while associations between TIMP-1 and ordinal and interval variables (ECOG performance status, age at randomization, number of metastatic sites, and disease-free interval) were evaluated by the Wilcoxon rank sum test.

Time to progression (TTP) was the primary endpoint in the original trial [28] as well as in this biomarker sub-study, and secondary endpoints were overall survival (OS) and response rate (RR). TTP was measured from date of randomization to date of documented progression with censoring at date of last visit or death. OS was calculated from date of randomization to date of death with censoring for surviving patients at last visit date. Time-to-event endpoints (TTP and OS) were estimated by the Kaplan-Meier method, and associations to TIMP-1 status were evaluated by the log-rank test. Analyses of TIMP-1 were done unadjusted as well as adjusted for preselected covariates in multivariate Cox proportional hazards models. The preselected covariates were those found to be significant in the previous analysis of the main study [28] and in a subsequent correlative sub-study including PAM50 intrinsic subtype [29]: treatment regimen, disease type (visceral vs. nonvisceral), stage of disease, performance status, number of metastatic sites, and PAM50 intrinsic subtype. The adjusted model was further stratified for previous chemotherapy [28]. The assumption of proportional hazards was assessed by Schoenfeld residuals. Subgroup analyses were done to assess whether treatment effects on TTP and OS differed according to TIMP-1 status or the levels of preselected variables. In addition, explorative analysis of treatment effect heterogeneity according to the combined TIMP-1 and PAM50 intrinsic subtype status (TIMP-1 positive and non-basal-like vs. TIMP-1 negative and/or basal-like) was evaluated. The multivariate Cox proportional hazards model was extended by one interaction term at a time. The interaction terms were tested using the Wald test and results were given in a Forest plot. RR was evaluated for patients with measurable disease. The overall RR was defined as a complete or partial response according to RECIST criteria, version 1.0. RRs were compared by using Fisher’s exact test.

Statistical analyses were conducted using SAS version 9.2 software package (SAS Institute, Cary, NC, USA). All statistical tests were two sided, and *P* < 0.05 considered statistically significant. Reporting Recommendations for Tumor Marker Prognostic Studies (REMARK) were adhered to wherever applicable [32]. The design of the study was prospective-retrospective as described by Simon et al. [33].

## Results

### TIMP-1 IHC staining

**Figure 1:**
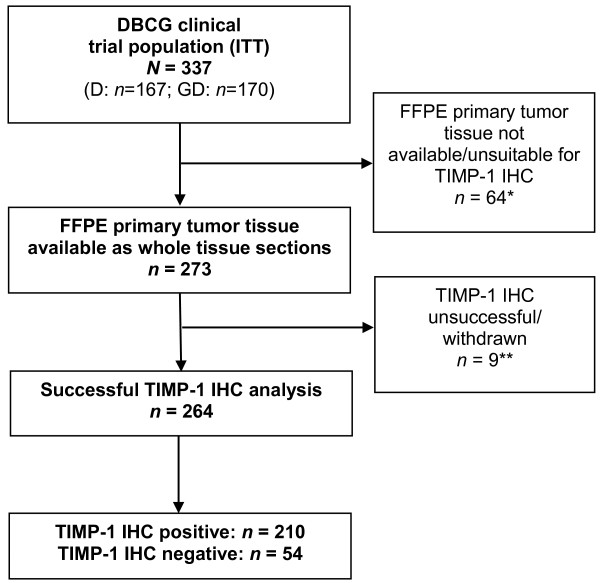
**CONSORT diagram.** *Tissue samples were unavailable/unsuitable for one of the following reasons: archival tissue not available (n = 39), no tumor cells in available samples (n = 13), only needle biopsies available (n = 12). **Tissue samples were withdrawn for one of the following reasons: IHC analysis done on metastasis only (n = 3), patients with bilateral cancer (n = 4), IHC analysis unsuccessful (n = 2). Abbreviations: D = docetaxel; DBCG = Danish Breast Cancer Cooperative Group; FFPE = formalin-fixed, paraffin-embedded; GD = gemcitabine plus docetaxel; IHC = immunohistochemical staining; TIMP-1 = tissue inhibitor of metalloproteinases-1.

Archival FFPE primary tumor tissue blocks from patients enrolled in the trial were retrospectively collected between January 2006 and December 2010 from study sites and centrally stored. The original trial recruited 337 participants, and for the present study a total of 273 tumors were available for TIMP-1 analysis (Figure 1). TIMP-1 IHC staining was successful in 264 patients. The 264 TIMP-1 assessable patients differed significantly from the 73 non-assessable patients (P < 0.05) with regard to stage of disease, (neo)adjuvant chemotherapy, adjuvant hormonal therapy, and adjuvant radiation therapy (Table 1). Among the assessable 264 patients, 210 (80%) had a TIMP-1-positive tumor. TIMP-1 status was not correlated with any of the baseline characteristics (Table 2).

**Table 1:** Patient demographics, disease characteristics, and prior therapy

		**Included**			**Excluded**		
**Characteristics**	**No.**		**(%)**	**No.**		**(%)**	**P**^**d**^
No. of patients	264			73			
Regimen							0.36
Docetaxel and Gemcitabine	137		(51.9)	33		(45.2)	
Docetaxel	127		(48.1)	40		(54.8)	
Median age at randomization, years		59			57		0.57^e^
Range		30-75			36-73		
ECOG performance status							0.84
0-1	223		(84.5)	65		(89.0)	
2	32		(12.1)	8^a^		(11.0)	
Unknown	9		(3.4)	0		(0.0)	
Stage of disease							0.049
Locally advanced	22		(8.3)	12		(16.4)	
Metastatic	242		(91.7)	61		(83.6)	
No. of metastatic sites							0.74
1	78		(29.5)	18		(24.7)	
2	93		(35.2)	27		(37.0)	
≥ 3	93		(35.2)	28		(38.4)	
Type of metastatic site							
Visceral	150		(56.8)	43		(58.9)	0.79
Lung	78		(29.5)	25		(34.2)	
Liver	99		(37.5)	27		(37.0)	
Non-visceral	114		(43.2)	30		(41.1)	
Bone	175		(66.3)	41		(56.2)	
Hormone receptor status							0.76
Positive	190		(72.0)	49		(67.1)	
Negative	70		(26.5)	20		(27.4)	
Unknown	4		(1.5)	4		(5.5)	
*HER2* status^b^							0.77
Normal/deletion	212		(80.3)	20		(27.4)	
Amplification	37		(14.0)	4		(5.5)	
Unknown	15		(5.7)	49		(67.1)	
PAM50 subtype							0.18
Luminal A	78		(29.5)	6		(8.2)	
Luminal B	94		(35.6)	3		(4.1)	
Basal-like	40		(15.2)	3		(4.1)	
HER2-enriched	46		(17.4)	0		(0.0)	
Unknown	6		(2.3)	61		(83.6)	
Prior chemotherapy							
Total	188		(71.2)	45		(61.6)	0.15
(Neo)adjuvant	127		(48.1)	17		(23.3)	0.0002
Anthracycline	71		(26.9)	11		(15.1)	
Non-anthracycline	56		(21.2)	6		(8.2)	
Locally advanced/metastatic	102		(38.6)	31		(42.5)	0.20
Anthracycline	86		(32.6)	30		(41.1)	
Non-anthracycline	16		(6.1)	1		(1.4)	
Hormonal therapy							
Total	173		(65.5)	41		(56.2)	0.17
Adjuvant	119		(45.1)	19		(26.0)	0.006
Locally advanced/metastatic	120		(45.5)	37		(50.7)	0.43
Radiation therapy	157		(59.5)	22		(30.1)	<0.0001
Disease-free interval, months^c^							
Median		31			22		0.12^e^
Range		0-250			0-231		

^a^Including one patient ECOG performance status 3.

^b^Retrospective analysis, primary tumor only.

^c^Time interval from diagnosis of primary cancer to recurrence.

^d^Fisher's exact test, unknown values excluded from tests.

^e^Wilcoxon test.

*Abbreviations*: *ECOG* Eastern Cooperative Oncology Group, *HER2* Human epidermal growth factor receptor 2.

**Table 2:** Association between TIMP-1 status and patient demographics, disease characteristics, and prior therapy

		**TIMP-1 negative**			**TIMP-1 positive**		
**Characteristics**	**No.**		**(%)**	**No.**		**(%)**	**P**^**c**^
No. of patients	54			210			
Regimen							0.36
Docetaxel and Gemcitabine	25		(46.3)	112		(53.3)	
Docetaxel	29		(53.7)	98		(46.7)	
Median age at randomization, years		60			59		0.79^e^
Range		37-74			30-75		
ECOG performance status							0.91
0-1	47		(87.0)	176		(83.8)	
2	6		(11.1)	26		(12.4)	
Unknown	1		(1.9)	8		(3.8)	
Stage of disease							0.58
Locally advanced	3		(5.6)	19		(9.0)	
Metastatic	51		(94.4)	191		(91.0)	
No. of metastatic sites							0.40
1	12		(22.2)	66		(31.4)	
2	22		(40.7)	71		(33.8)	
≥ 3	20		(37.0)	73		(34.8)	
Type of metastatic site							
Visceral	31		(57.4)	119		(56.7)	1.00
Lung	17		(31.5)	61		(29.0)	0.87
Liver	18		(33.3)	81		(38.6)	0.44
Non-visceral	23		(42.6)	91		(43.3)	
Bone	33		(61.1)	142		(67.6)	0.42
Hormone receptor status							0.30
Positive	42		(77.8)	148		(70.5)	
Negative	11		(20.4)	59		(28.1)	
Unknown	1		(1.9)	3		(1.4)	
*HER2* status^a^							0.66
Normal/deletion	40		(74.1)	172		(81.9)	
Amplification	8		(14.8)	29		(13.8)	
Unknown	6		(11.1)	9		(4.3)	
PAM50 subtype							0.89
Luminal A	15		(27.8)	63		(30.0)	
Luminal B	20		(37.0)	74		(35.2)	
Basal-like	7		(13.0)	33		(15.7)	
HER2-enriched	11		(20.4)	35		(16.7)	
Unknown	1		(1.9)	5		(2.4)	
Prior chemotherapy							
Total	37		(68.5)	151		(71.9)	0.62
(Neo)adjuvant	23		(42.6)	104		(49.5)	0.45
Anthracycline	14		(25.9)	57		(27.1)	
Non-anthracycline	9		(16.7)	47		(22.4)	
Locally advanced/metastatic	21		(38.9)	81		(38.6)	1.00
Anthracycline	15		(27.8)	71		(33.8)	
Non-anthracycline	6		(11.1)	10		(4.8)	
Hormonal therapy							
Total	36		(66.7)	137		(65.2)	0.87
Adjuvant	24		(44.4)	95		(45.2)	1.00
Locally advanced/metastatic	27		(50.0)	93		(44.3)	0.54
Radiation therapy	33		(61.1)	124		(59.0)	0.88
Disease-free interval, months^b^							
Median		36			31		0.41^e^
Range		0-224			0-250		

^a^Retrospective analysis, primary tumor only.

^b^Time interval from diagnosis of primary cancer to recurrence.

^c^Fisher's exact test, unknown values excluded from tests.

^e^Wilcoxon test.

*Abbreviations*: *ECOG* Eastern Cooperative Oncology Group, *HER2* Human epidermal growth factor receptor 2, *TIMP-1* Tissue inhibitor of metalloproteinases-1.

### Prognosis and response rates

**Figure 2:**
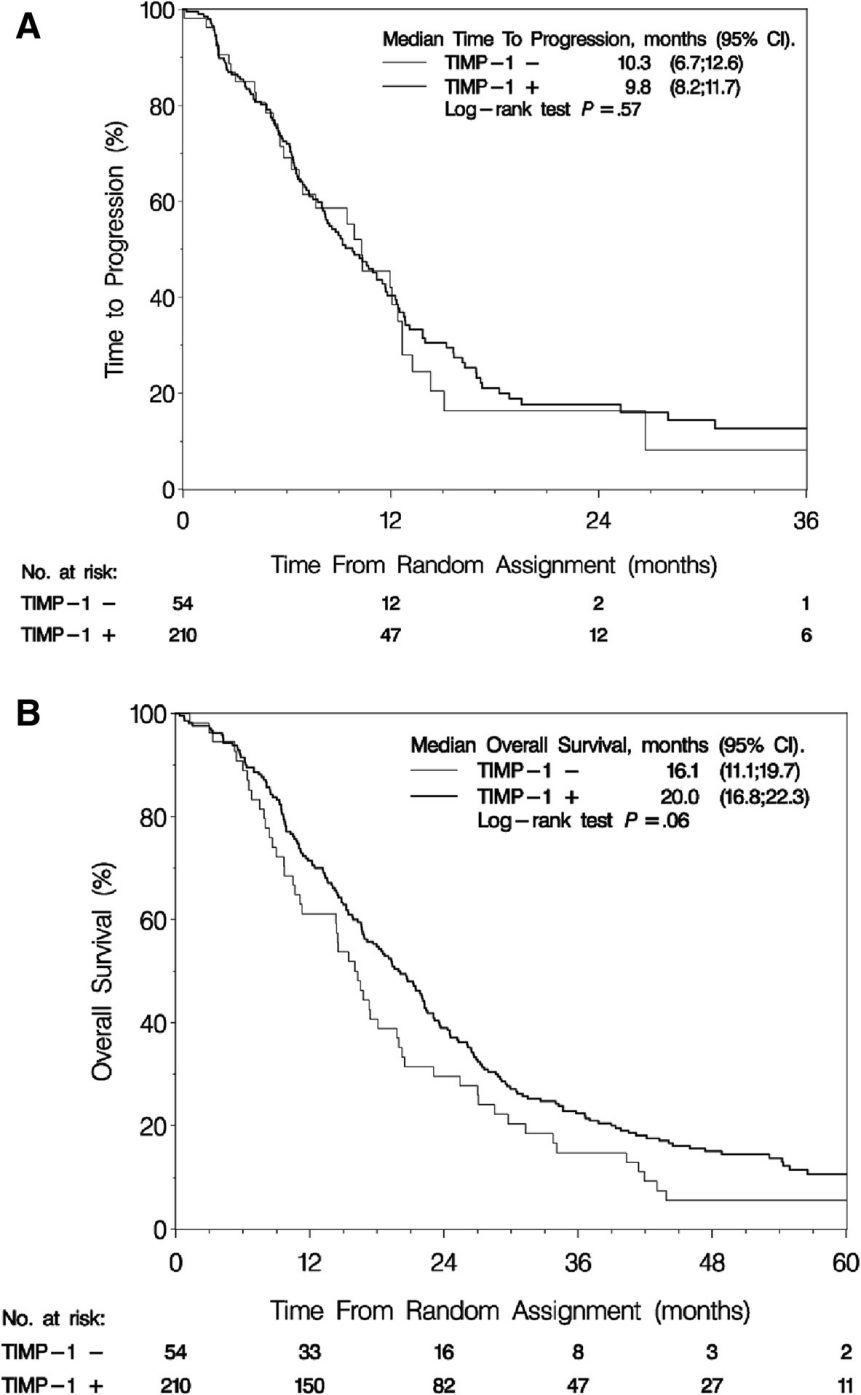
**Time to progression and overall survival according to TIMP-1 status.** Kaplan-Meier curve for **(A)** time to progression (165 events) and **(B)** overall survival (240 events) for all 264 advanced breast cancer patients treated with gemcitabine plus docetaxel or docetaxel alone (study arms combined) according to TIMP-1 immunohistochemical staining status. Abbreviations: CI = confidence interval; TIMP-1 = tissue inhibitor of metalloproteinases-1.

In univariate analyses, TIMP-1 status was not associated with TTP or OS, however, there was a non-significant trend that TIMP-1 positive patients had increased OS (P = 0.06) (Figure 2) (Table 3).

**Table 3:** **Cox univariate models for time to progression and overall survival**^**a**^

			**Time to progression**				**Overall survival**		
**Risk factor**	**n**	**HR**	**95% CI**		**P**	**HR**	**95% CI**		**P**
TIMP-1 (positive vs. negative)	264	0.90	(0.61-	1.31)	0.57	0.742	(0.55-	1.01)	0.06

^a^Models not stratified.

*Abbreviations*: *CI* Confidence interval, *HR* Hazard ratio, *TIMP-1* Tissue inhibitor of metalloproteinases-1.

Treatment effects in the study population were similar to those found in the original study [28] (TTP: adjusted HR = 0.60; 95% CI 0.42-0.84; P = 0.003; OS: adjusted HR = 0.88; 95% CI 0.67-1.15; P = 0.34) (Table 4).

**Table 4:** **Cox multivariable models for time to progression and overall survival**^**a**^

			**Time to progression**				**Overall survival**		
**Risk factor**	**n**	**HR**	**95% CI**		**P**	**HR**	**95% CI**		**P**
Regimen (GD vs. D)		0.60	(0.42-	0.84)	0.003	0.88	(0.67-	1.15)	0.34
TIMP-1 (positive vs. negative)		0.82	(0.55-	1.21)	0.31	0.71	(0.52-	0.98)	0.03
PAM50					0.007				0.0001
Luminal A	78	0.68	(0.45-	1.04)	0.08	0.78	0.56	1.08	0.14
Luminal B	100^b^	1.00	referent			1.00	referent		
Basal-like	40	1.75	(1.05-	2.92)	0.03	2.39	(1.56-	3.64)	<0.0001
HER2-enriched	46	1.30	(0.82-	2.07)	0.27	1.25	(0.85-	1.84)	0.25
Visceral disease (yes vs. no)		1.60	(1.07-	2.39)	0.02	1.13	(0.84-	1.53)	0.42
Stage of disease (locally advanced vs. metastatic)		1.96	(1.02-	3.76)	0.04	0.72	(0.42-	1.24)	0.23
Metastatic sites (3+ vs. 1-2)		1.24	(0.86-	1.77)	0.25	1.52	(1.13-	2.04)	0.01
ECOG performance status (2 vs. 0-1)		1.19	(0.85-	1.67)	0.31	1.47	(1.12-	1.93)	0.006

^a^Models stratified for previous chemotherapy (none, n = 76; adjuvant, n = 86; locally advanced or metastatic, n = 102).

^b^Including 6 records with missing value of PAM50 molecular subtype.

*Abbreviations*: *CI* Confidence interval, *D* Docetaxel, *ECOG* Eastern Cooperative Oncology Group,* G* Gemcitabine, *HER2* Human epidermal growth factor receptor 2, *HR* Hazard ratio, *TIMP-1* Tissue inhibitor of metalloproteinases-1.

In Cox multivariate analysis adjusted for regimen, PAM50 subtype, presence of visceral disease, stage of disease, number of metastatic sites, and ECOG performance status, TIMP-1 positivity was an independent prognostic factor with regard to OS (adjusted HR = 0.71; 95% CI 0.52-0.98; P = 0.03) but not for TTP (adjusted HR = 0.82; 95% CI 0.55-1.21; P = 0.31) (Table 4).

RR among the 164 patients with measurable disease did not differ significantly according to TIMP-1 status (Table 5).

**Table 5:** **Best overall response**^**a **^**by TIMP-1 protein status**^**b**^

	**TIMP-1 Positive**		**TIMP-1 Negative**	
**Response**	**No.**	**(%)**	**No.**	**(%)**
CR	4	(3.0)	2	(6.3)
PR	46	(34.8)	9	(28.1)
Total responses	50	(37.9)	11	(34.4)
(95% CI)	(24.2 to 40.8)			(30.7 to 69.4)
SD	56	(42.4)	15	(46.9)
PD	15	(11.4)	5	(15.6)
Unknown	11	(8.3)	1	(3.1)
Total	132		32	

^a^Measurable disease (n = 164).

^b^Total responses, Fisher's exact test P =0.84.

*Abbreviations*: *CI* Confidence interval, *CR* Complete response, *PD* Progressive disease, *PR* Partial response, *SD* Stable disease, *TIMP-1* Tissue inhibitor of metalloproteinases-1.

### TIMP-1 subgroup analysis

**Figure 3:**
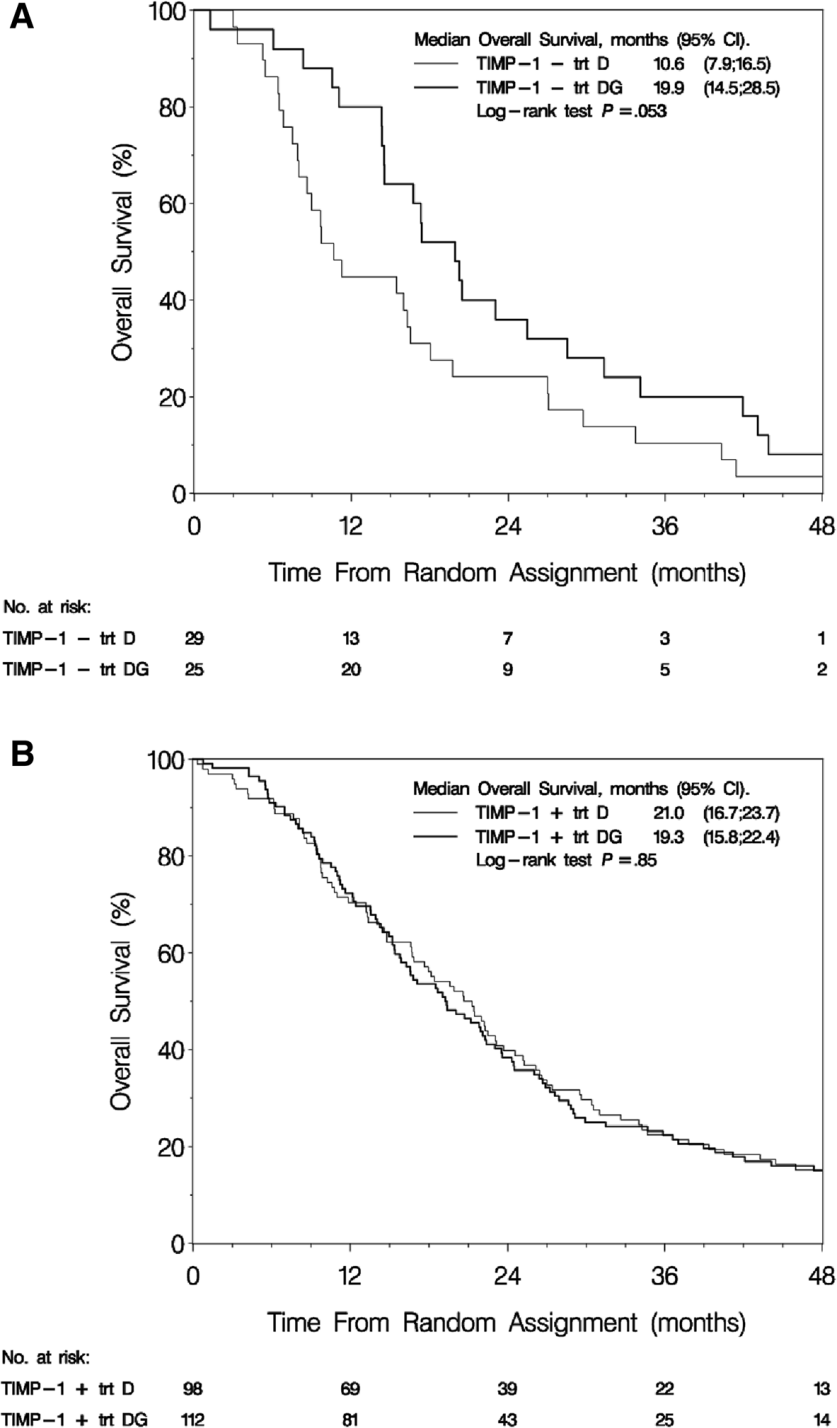
**Overall survival according to TIMP-1 status and treatment.** Kaplan-Meier curve for overall survival for **(A)** TIMP-1 negative patients and **(B)** TIMP-1 positive patients according to treatment allocation. Abbreviations: CI = confidence interval; D = docetaxel; DG = docetaxel plus gemcitabine; TIMP-1 = tissue inhibitor of metalloproteinases-1; trt = treatment.

In unadjusted analysis an estimated gain in median OS of nine months was seen in the doublet arm compared to the monotherapy arm for patients with a TIMP-1 negative tumor (GD, median OS: 19.9 months, 95% CI = 14.5-28.5; D, median OS: 10.6 months, 95% CI = 7.9-16.5, log rank p = 0.053) (Figure 3A). No difference in OS according to regimen was detected in patients with TIMP-1 positive tumors (Figure 3B).

**Figure 4:**
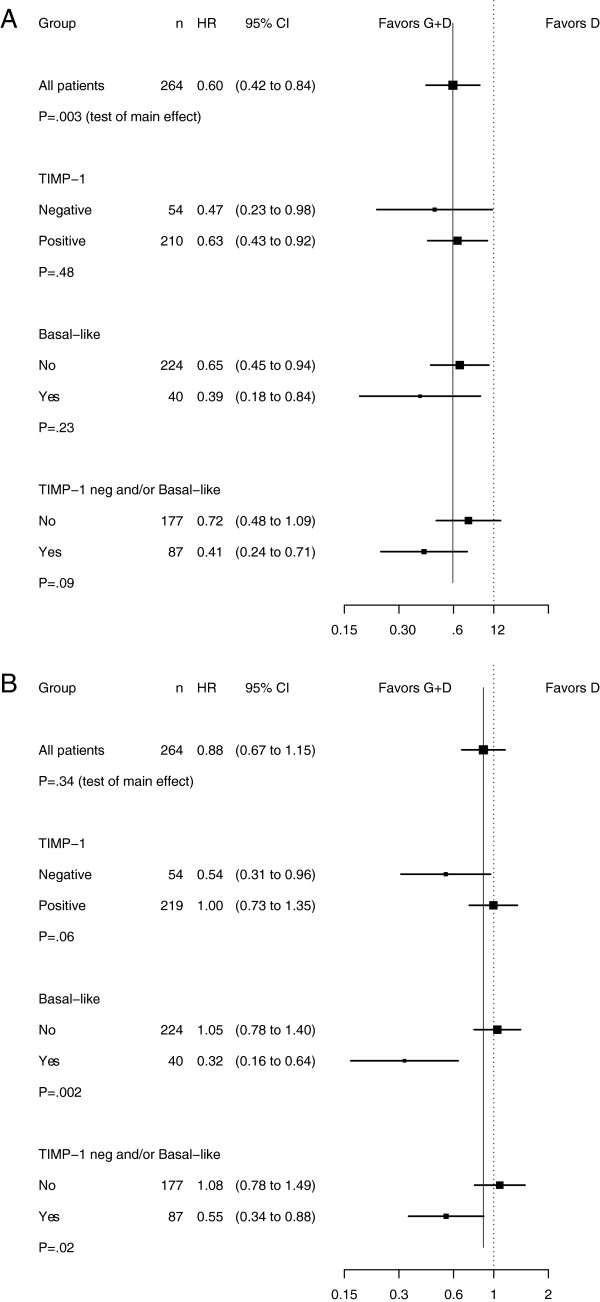
**Subgroup analyses.** Forest plots illustrating hazard ratio (HR) estimates of treatment effect with 95% confidence intervals (CI) for **(A)** time to progression and **(B)** overall survival comparison between patients with TIMP-1 negative and TIMP-1 positive tumors, basal-like and non-basal-like tumors, TIMP-1 negative and/or basal-like (G responsive) and TIMP-1 positive and non-basal-like tumors (G nonresponsive). Abbreviations: D = docetaxel; G = gemcitabine; TIMP-1 = tissue inhibitor of metalloproteinases-1.

In multivariate Cox regression analyses adjusted for the preselected covariates no interaction was demonstrated between TIMP-1 status and treatment regimens for TTP (Figure 4A). For OS a borderline significant interaction was demonstrated such that GD improved OS compared to D in patients with TIMP-1 negative tumors (Figure 4B P_interaction_ = 0.06).

### Explorative analysis of gemcitabine responsive subgroup

In this trial we have previously demonstrated a substantial reduction in mortality by GD compared to D in patients with basal-like tumors [29]. In the current study PAM50 intrinsic subtype remained an independent factor in the multivariate analysis with TIMP-1. To compare TIMP-1 as a single marker against the combination of TIMP-1 and PAM50 intrinsic subtype status further explorative analyses were conducted. A total of 87 (33%) patients were classified as G responsive (e.g. basal-like subtype and/or lack of TIMP-1 immunoreactivity). In Kaplan-Meier analysis patients classified as G responsive had a significant improvement in both TTP (GD, median TTP: 10.3 months, 95% CI = 7.7-12.6; D, median TTP: 6.2 months, 95% CI = 4.1-9.5) and OS (GD, median OS: 17.4 months, 95% CI = 14.4-20.7; D, median OS: 10.0 months, 95% CI = 7.9-15.5) if treated with GD compared with D. Multivariate analysis adjusted for patient and tumor characteristics confirmed these results for OS (P_interaction_ = 0.02, Figure 4B) but not for TTP (P_interaction_ = 0.09, Figure 4A).

## Discussion

In the present study, TIMP-1 cancer cell immunoreactivity was associated with a reduction in mortality but not with a reduction in TTP events (primary endpoint). Furthermore, in patients without TIMP-1 cancer cell immunoreactivity, we identified a 46% relative reduction in mortality from the addition of G to D compared to single agent D, although this difference was not statistically significant (P_interaction_ = 0.06).

The majority of breast cancer studies on TIMP-1 and association with prognosis and response to chemotherapy have focused on patients receiving adjuvant chemotherapy [2,8,18,20,34], whereas only two studies have included patients with advanced breast cancer [35,36]. These two studies both measured TIMP-1 levels in the primary tumors using an enzyme-linked immunosorbent assay-based approach and included patients receiving cyclophosphamide/methotrexate/5-fluorouracil or anthracycline-based chemotherapy. In the exploratory study by Schrohl et al. [36] results suggested that patients with high levels of TIMP-1 are less responsive to chemotherapy, but the study did not include an analysis of OS. On the other hand, the second study by Klintman et al. [35] which supports the association of TIMP-1 with objective response to chemotherapy, did not find TIMP-1 to be associated with either progression free survival (PFS) or OS. The results presented in this manuscript suggest that TIMP-1 protein expression in cancer cells in the primary tumor evaluated by IHC is associated with improved OS for advanced breast cancer patients receiving D or GD. The use of different methodologies and regimens does compromise the comparability of studies, and thus, the prognostic significance of TIMP-1 in advanced breast cancer remains unclear.

The predictive value of TIMP-1 in relation to chemotherapy has been evaluated in a few studies [2,8,20,21] but to our knowledge the relationship between TIMP-1 and the effect of G has not been addressed previously in a preclinical or clinical setting. In the present study we found a borderline statistically significant difference in OS favoring GD over D alone for patients evaluated as TIMP-1 negative. TIMP-1 negative patients receiving GD increased their survival by almost 50% to a median survival of 19.9 months compared to 10.6 months for patients receiving D only. Whether this survival benefit can be attributed to the addition of G specifically or whether our findings suggest a more general effect of chemotherapy doublets/D containing doublets in advanced breast cancer remains speculative and hypothesis generating. We were, however, able to identify a subgroup of patients that derived clinically meaningful benefit from combination chemotherapy as compared to single agent chemotherapy using TIMP-1 immunoreactivity although the results did not reach statistical significance (P = 0.06).

Preclinical data suggest that the anti-apoptotic functions of TIMP-1 are mediated through the phosphatidylinositol 3-kinase (PI3K)/Akt survival pathway [15,37] and TIMP-1 in this manner protects cancer cells from the effects of chemotherapy and hence cancer cells lacking TIMP-1 would remain sensitive to chemotherapy. Interestingly, the PI3K/Akt pathway has previously been suggested to be implicated in gemcitabine resistance [38]. Other preclinical findings suggest that TIMP-1 modifies proliferation by direct regulation of the cell cycle by arresting cells in G1 phase [39]. This could possibly alter the response to cell cycle specific drugs such as G, as the cytotoxic effect of G is associated primarily with specific inhibition of cells in the S phase [25,40]. Furthermore, low levels of TIMP-1 have been associated with sensitivity to anthracycline- [2,8,20,21] and irinotecan-containing therapy [41]. Since anthracyclines are topoisomerase-2 inhibitors and irinotecan is a topoisomerase-1 inhibitor, TIMP-1 may particularly interact with topoisomerase inhibitors. G has been shown to poison topoisomerase I [42,43], suggesting that TIMP-1 can influence the effect of G through this enzyme. Taken together, these findings, along with the results obtained in the present study, make a ‘broader’ predictive role of TIMP-1 in the treatment of breast cancer likely.

Clinical trials in advanced breast cancer and other solid tumors often use TTP and PFS as primary endpoints and surrogate markers for OS [44,45]. However, an increase in TTP or PFS does not always translate into a survival benefit. On the other hand, the results presented here demonstrate that neither the prognostic impact of TIMP-1 nor the trend of interaction between TIMP-1 status and treatment regarding OS were reflected in the analysis of the primary endpoint TTP. A reason for this discrepancy could be related to the nine-weekly disease-evaluations used in the current trial, which may have generated a systematic bias and less precise evaluation of TTP as compared to the OS endpoint where the date of death is exact [44]. Given that both tumor promoting and inhibitory effects have been described for TIMP-1 [12-16], and that cellular TIMP-1 function depends on the surrounding microenvironment [46,47] the discrepancy between endpoints could also be related to a biological role of TIMP-1 such that the role of TIMP-1 is different in the case of localized cancer compared to the advanced disease setting, perhaps by preventing further spread of metastasis at this stage.

We have previously shown in this trial a differential benefit from the addition of G to D in patients with a basal-like intrinsic subtype classified by the PAM50 assay [29]. The fact that TIMP-1 status was not associated with PAM50 intrinsic subtype, suggests that TIMP-1 negative status and the basal-like subtype characterize two biologically distinct mechanisms of a possible G responsiveness. For comparison, in an exploratory analysis, we combined TIMP-1 and PAM50 subtype into a panel and classified patients as G responsive if tumors lacked TIMP-1 immunoreactivity and/or were characterized as basal-like, or otherwise as G nonresponsive. Using this panel 33% of the patients could be classified as G responsive compared with 20% and 15% using TIMP-1 status or PAM50 intrinsic subtype classification, respectively. The benefit from GD compared to D was considerably larger in patients with a G responsive profile, and this heterogeneity was confirmed by a statistically significant test of interaction between this panel and treatment for OS. Thus, the PAM50 intrinsic subtype/TIMP-1 protein status panel identifies the patients most likely to benefit from GD compared to D in terms of OS, and additionally, this panel appears to identify and separate two-thirds of the patients unlikely to derive any benefit from this combination therapy.

A strength of this study is that data from 78% of the patients enrolled in a randomized phase III clinical trial with long-term follow-up were available for analysis. In addition, we applied a previously validated assay for TIMP-1 immunoreactivity. On the other hand this study also has some potential limitations. The statistical power was limited due to the small population size, especially under-powering the results of the subgroup analysis. Another limitation is the fact that we did not include TIMP-1 staining of stromal cells in the analyses. It has been suggested that TIMP-1 may be produced by stromal cells and eventually become absorbed by breast cancer cells [48], and an association between stromal TIMP-1 expression status and progression of cancer has been reported [49,50], although not consistently [51,52]. In the current study TIMP-1 expression was exclusively evaluated in breast cancer cells, and the outcome might have been different if TIMP-1 had been evaluated in stromal cells or in stromal as well as cancer cells. Furthermore, TIMP-1 analysis was performed on primary tumor tissue and not on corresponding metastases. We cannot be sure that the status of TIMP-1 will be the same in the metastases as in the primary tumor since the molecular portrait of the tumor could have changed pronouncedly in the period in between primary diagnosis and recurrence, especially as a majority of the patients received prior treatment. A substantial discordance in ER and *HER2* status between primary and metastatic tumor tissue has been reported [53-55], and a study has demonstrated differences in the immunoreactivity of TIMP-1 in primary breast tumor tissue and the corresponding axillary lymph node metastasis [56]. TIMP-1 expression in primary and metastatic tumor tissue have not been compared, but usage of metastatic tumor tissue could potentially have had an impact on the results.

## Conclusions

In summary, this retrospective analysis applied to a prospective clinical trial demonstrated that TIMP-1 status appears to contain an independent prognostic value regarding overall survival in patients with advanced breast cancer receiving chemotherapy. We demonstrated that OS was almost doubled for patients with a TIMP-1 negative status receiving the combination regimen as compared to single agent chemotherapy. However, the test for interaction between TIMP-1 status and treatment did only reach borderline significance. Furthermore, we could not show a similar reduction in TTP events, the pre-specified primary endpoint of this study. The results presented here need further validation in order to obtain convincing evidence that TIMP-1 may be used as a predictive marker to direct the use of G in combination with D for patients with advanced breast cancer.

## Abbreviations

D: Docetaxel; DBCG: Danish Breast Cancer Cooperative Group; ECOG: Eastern Cooperative Oncology Group; FFPE: Formalin-fixed, paraffin-embedded; G: Gemcitabine; G1: Growth phase 1; HER2: Human epidermal growth factor receptor 2; IHC: Immunohistochemical staining; OS: Overall survival; PFS: Progression free survival; PI3K: Phosphatidylinositol 3-kinase; REMARK: Reporting recommendations for tumor marker prognostic studies; RR: Response rate; TIMP-1: Tissue inhibitor of metalloproteinases-1; TTP: Time to progression.

## Competing interests

The authors declare that they have no competing interests.

## Authors’ contributions

BE, EB, NB, CB and CLTJ made contributions to conception and design of the study. DLN and BE provided access to clinical data on study material, and EB and CLTJ collected the tissue samples. AB performed the immunohistochemical staining. Evaluation of the stainings was performed by EB, AB, and CLTJ. KDB performed the statistical analysis. All authors contributed to analysis and interpretation of data. CLTJ and CB drafted the manuscript, and all authors contributed to the manuscript preparation and in revising the manuscript critically. All authors read and approved the final manuscript, and are accountable for all aspects of the work.

## Pre-publication history

The pre-publication history for this paper can be accessed here:

http://www.biomedcentral.com/1471-2407/14/360/prepub

## References

[B1] Cardoso F Bedard PL Winer EP Pagani O Senkus-Konefka E Fallowfiled LL Kyriakides S Costa A Cufer T Albain KS International guidelines for management of metastatic breast cancer: combination vs sequential single-agent chemotherapy J Natl Cancer Inst 2009 101 1174 1181 19657108 10.1093/jnci/djp235 PMC2736293

[B2] Ejlertsen B Jensen MB Nielsen KV Balslev E Rasmussen BB Willemoe GL Hertel PB Knoop AS Mouridsen HT Brunner N HER2, TOP2A, and TIMP-1 and responsiveness to adjuvant anthracycline-containing chemotherapy in high-risk breast cancer patients J Clin Oncol 2010 28 984 990 20038724 10.1200/JCO.2009.24.1166

[B3] Henriksen KL Rasmussen BB Lykkesfeldt AE Moller S Ejlertsen B Mouridsen HT An ER activity profile including ER, PR, Bcl-2 and IGF-IR may have potential as selection criterion for letrozole or tamoxifen treatment of patients with advanced breast cancer Acta Oncol 2009 48 522 531 19173092 10.1080/02841860802676383

[B4] Knoop AS Knudsen H Balslev E Rasmussen BB Overgaard J Nielsen KV Schonau A Gunnarsdottir K Olsen KE Mouridsen H Ejlertsen B Danish Breast Cancer Cooperative G Retrospective analysis of topoisomerase IIa amplifications and deletions as predictive markers in primary breast cancer patients randomly assigned to cyclophosphamide, methotrexate, and fluorouracil or cyclophosphamide, epirubicin, and fluorouracil: Danish Breast Cancer Cooperative Group J Clin Oncol 2005 23 7483 7490 16234514 10.1200/JCO.2005.11.007

[B5] Nielsen KV Muller S Moller S Schonau A Balslev E Knoop AS Ejlertsen B Aberrations of ERBB2 and TOP2A genes in breast cancer Mol Oncol 2010 4 161 168 19945923 10.1016/j.molonc.2009.11.001 PMC5527893

[B6] Nielsen KV Ejlertsen B Moller S Jorgensen JT Knoop A Knudsen H Mouridsen HT The value of TOP2A gene copy number variation as a biomarker in breast cancer: update of DBCG trial 89D Acta Oncol 2008 47 725 734 18465341 10.1080/02841860801995396

[B7] Perez EA Impact, mechanisms, and novel chemotherapy strategies for overcoming resistance to anthracyclines and taxanes in metastatic breast cancer Breast Cancer Res Treat 2009 114 195 201 18443902 10.1007/s10549-008-0005-6

[B8] Willemoe GL Hertel PB Bartels A Jensen MB Balslev E Rasmussen BB Mouridsen H Ejlertsen B Brunner N Lack of TIMP-1 tumour cell immunoreactivity predicts effect of adjuvant anthracycline-based chemotherapy in patients (n = 647) with primary breast cancer. A Danish Breast Cancer Cooperative Group Study Eur J Cancer 2009 45 2528 2536 19535243 10.1016/j.ejca.2009.05.029

[B9] Goldhirsch A Wood WC Coates AS Gelber RD Thülimann B Senn H-J Members P Strategies for subtypes- dealing with the diversity of breast cancer: highlights of the St Gallen international expert consensus on the primary therapy of early breast cancer 2011 Ann Oncol 2011 22 1736 1747 21709140 10.1093/annonc/mdr304 PMC3144634

[B10] Harris L Fritsche H Mennel R Norton L Ravdin P Taube S Somerfield MR Hayes DF Bast RC Jr American society of clinical O: American society of clinical oncology 2007 update of recommendations for the use of tumor markers in breast cancer J Clin Oncol 2007 25 5287 5312 17954709 10.1200/JCO.2007.14.2364

[B11] Subramaniam DS Isaacs C Utilizing prognostic and predictive factors in breast cancer Curr Treat Options Oncol 2005 6 147 159 15717996 10.1007/s11864-005-0022-1

[B12] Chirco R Liu XW Jung KK Kim HR Novel functions of TIMPs in cell signaling Cancer Metastasis Rev 2006 25 99 113 16680576 10.1007/s10555-006-7893-x

[B13] Jiang Y Goldberg ID Shi YE Complex roles of tissue inhibitors of metalloproteinases in cancer Oncogene 2002 21 2245 2252 11948407 10.1038/sj.onc.1205291

[B14] Stetler-Stevenson WG Tissue inhibitors of metalloproteinases in cell signaling: Metalloproteinase-independent biological activities Sci Signal 2008 1 re6 18612141 10.1126/scisignal.127re6 PMC2493614

[B15] Wurtz SO Schrohl AS Mouridsen H Brunner N TIMP-1 as a tumor marker in breast cancer–an update Acta Oncol 2008 47 580 590 18465326 10.1080/02841860802022976

[B16] Wurtz SO Schrohl AS Sorensen NM Lademann U Christensen IJ Mouridsen H Brunner N Tissue inhibitor of metalloproteinases-1 in breast cancer Endocr Relat Canc 2005 12 215 227 10.1677/erc.1.00719 15947098

[B17] Dechaphunkul A Phukaoloun M Kanjanapradit K Graham K Ghosh S Santos C Mackey JR Prognostic significance of tissue inhibitor of metalloproteinase-1 in breast cancer Int J Breast Canc 2012 2012 290854 10.1155/2012/290854 PMC3440855 22988515

[B18] Neri A Megha T Bettarini F Tacchini D Mastrogiulio MG Marrelli D Pinto E Tosi P Is tissue inhibitor of metalloproteinase-1 a new prognosticator for breast cancer? An analysis of 266 cases Hum Pathol 2012 43 1184 1191 22397869 10.1016/j.humpath.2011.09.018

[B19] Wu ZS Wu Q Yang JH Wang HQ Ding XD Yang F Xu XC Prognostic significance of MMP-9 and TIMP-1 serum and tissue expression in breast cancer Int J Cancer 2008 122 2050 2056 18172859 10.1002/ijc.23337

[B20] Hertel PB Tu D Ejlertsen B Jensen MB Balslev E Jiang S O'Malley FP Pritchard KI Shepherd LE Bartels A Brunner N Nielsen TO TIMP-1 in combination with HER2 and TOP2A for prediction of benefit from adjuvant anthracyclines in high-risk breast cancer patients Breast Cancer Res Treat 2012 132 225 234 22160637 10.1007/s10549-011-1896-1

[B21] Schrohl AS Look MP Meijer-van Gelder ME Foekens JA Brunner N Tumor tissue levels of Tissue Inhibitor of Metalloproteinases-1 (TIMP-1) and outcome following adjuvant chemotherapy in premenopausal lymph node-positive breast cancer patients: A retrospective study BMC Cancer 2009 9 322 19744322 10.1186/1471-2407-9-322 PMC2754488

[B22] Montero A Fossella F Hortobagyi G Valero V Docetaxel for treatment of solid tumours: a systematic review of clinical data Lancet Oncol 2005 6 229 239 15811618 10.1016/S1470-2045(05)70094-2

[B23] Heinemann V Xu Y-Z Chubb S Sen A Hertel LW Grindey GB Plunkett W Inhibition of ribonucleotide reduction in CCRF-CEM cells by 2',2'-difluorodeoxycytidine Mol Pharmacol 1990 38 567 572 2233693

[B24] Heinemann V Hertel LW Grindley GB Plunkett W Comparison of the cellular pharmacokinetics and toxicity of 2',2'-difluorodeoxycytidine and 1-beta-d-arabinofuranosylcytosine Cancer Res 1988 48 4024 4031 3383195

[B25] Huang P Chubb S Hertel LW Grindey GB Plunkett W Action of 2´,2´-difluorodeoxycytidine on DNA synthesis Cancer Res 1991 51 6110 6117 1718594

[B26] Comen EA Formier MN Algorithms for the treatment of patients with metastatic breast cancer and prior exposure to taxanes and anthracyclines Clin Breast Canc Suppl 2010 10 S7 S19 10.3816/CBC.2010.s.008 20805067

[B27] Saghir NS Tfayli A Hatoum HA Nachef Z Dinh P Awada A Treatment of metastatic breast cancer: State-of-the-art, subtypes and perspectives Crit Rev Oncol Hematol 2011 80 433 449 21330148 10.1016/j.critrevonc.2011.01.010

[B28] Nielsen DL Bjerre KD Jakobsen EH Cold S Stenbygaard L Sorensen PG Kamby C Moller S Jorgensen CL Andersson M Gemcitabine plus docetaxel versus docetaxel in patients with predominantly human epidermal growth factor receptor 2-negative locally advanced or metastatic breast cancer: a randomized, phase III study by the Danish Breast Cancer Cooperative Group J Clin Oncol 2011 29 4748 4754 22084374 10.1200/JCO.2010.33.9507

[B29] Jørgensen CLT Nielsen TO Bjerre C Liu S Wallden B Balslev E Nielsen DL Ejlertsen B PAM50 breast cancer intrinsic subtypes and effect of gemcitabine in advanced breast cancer patients Acta Oncol 2013 In press 10.3109/0284186X.2013.865076 24359601

[B30] Sorensen IV Fenger C Winther H Foged NT Lademann U Brunner N Usher PA Characterization of anti-TIMP-1 monoclonal antibodies for immunohistochemical localization in formalin-fixed, paraffin-embedded tissue J Histochem Cytochem 2006 54 1075 1086 16517973 10.1369/jhc.5A6896.2006 PMC3957804

[B31] Moller Sorensen N Dowell BL Stewart KD Jensen V Larsen L Lademann U Murphy G Nielsen HJ Brunner N Davis GJ Establishment and characterization of 7 new monoclonal antibodies to tissue inhibitor of metalloproteinases-1 Tumour Biol 2005 26 71 80 15870512 10.1159/000085588

[B32] McShane LM Altman DG Sauerbrei W Taube SE Gion M Clark GM Statistics Subcommittee of the NCIEWGoCD: REporting recommendations for tumour MARKer prognostic studies (REMARK) Eur J Cancer 2005 41 1690 1696 16043346 10.1016/j.ejca.2005.03.032

[B33] Simon RM Paik S Hayes DF Use of archived specimens in evaluation of prognostic and predictive biomarkers J Natl Cancer Inst 2009 101 1446 1452 19815849 10.1093/jnci/djp335 PMC2782246

[B34] Wurtz SO Moller S Mouridsen H Hertel PB Friis E Brunner N Plasma and serum levels of tissue inhibitor of metalloproteinases-1 are associated with prognosis in node-negative breast cancer: a prospective study Mol Cell Proteomics 2008 7 424 430 17998244 10.1074/mcp.M700305-MCP200

[B35] Klintman M Ornbjerg Wurtz S Christensen IJ Braemer Hertel P Ferno M Malmberg M Mouridsen H Cold F Schrohl AS Foekens JA Malmstrom P Brunner N Association between tumor tissue TIMP-1 levels and objective response to first-line chemotherapy in metastatic breast cancer Breast Cancer Res Treat 2010 121 365 371 19653096 10.1007/s10549-009-0483-1

[B36] Schrohl AS Meijer-van Gelder ME Holten-Andersen MN Christensen IJ Look MP Mouridsen HT Brunner N Foekens JA Primary tumor levels of tissue inhibitor of metalloproteinases-1 are predictive of resistance to chemotherapy in patients with metastatic breast cancer Clin Cancer Res 2006 12 7054 7058 17114213 10.1158/1078-0432.CCR-06-0950

[B37] Fu ZY Lv JH Ma CY Yang DP Wang T Tissue inhibitor of metalloproteinase-1 decreased chemosensitivity of MDA-435 breast cancer cells to chemotherapeutic drugs through PI3/AKT/NF-kB pathway Biomed Pharmacother 2011 65 163 167 21684102 10.1016/j.biopha.2011.02.004

[B38] Ng SSW Tsao M-S Chow S Hedley DW Inhibition of phosphatidylinositide 3-kinase enhances gemcitabine-induced apoptosis in human pancreatic cancer cells Cancer Res 2000 60 5451 11034087

[B39] Taube ME Liu X-W Fridman R Kim H-RC TIMP-1 regulation of cell cycle in human breast epithelial cells via stabilization of p27 protein Oncogene 2006 25 3041 3048 16407831 10.1038/sj.onc.1209336

[B40] Hertel LW Boder GB Kroin JS Rinzel SM Poore GA Todd GC Grindey GB Evaluation of the antitumor activity of gemcitabine (2´,2´-difluoro-2´-deoxycytidine Cancer Res 1990 50 4417 2364394

[B41] Sorensen NM Bystrom P Christensen IJ Berglund A Nielsen HJ Brunner N Glimelius B TIMP-1 is significantly associated with objective response and survival in metastatic colorectal cancer patients receiving combination of irinotecan, 5-fluorouracil, and folinic acid Clin Cancer Res 2007 13 4117 4122 17634538 10.1158/1078-0432.CCR-07-0186

[B42] Gmeiner WH Yu S Pon RT Pourquier P Pommier Y Structural basis for topoisomerase I inhibition by nucleoside analogs Nucleosides Nucleotides Nucleic Acids 2003 22 653 658 14565246 10.1081/NCN-120022604

[B43] Pourquier P Gioffre C Kohlhagen G Urasaki Y Goldwasser F Hertel LW Yu S Pon RT Gmeiner WH Pommier Y Gemcitabine (2´,2´-difluoro-2´-deoxycytidine), an antimetabolite that poisons topoisomerase I Clin Cancer Res 2002 8 2499 2504 12171875

[B44] Burzykowski T Buyse M Piccart-Gebhart M Sledge G Carmichael J Lûck H-J Mackey JR Nabholtz J-M Paridaens R Biganzoli L Jassem J Beontenbal M Bonneterre J Chan S Basaran GA Therasse P Evaluation of tumor response, disease control, progression-free survival, and time to progression as potential surrogate end points in metastatic breast cancer J Clin Oncol 2008 26 1987 1992 18421050 10.1200/JCO.2007.10.8407

[B45] Sherrill B Amonkar M Wu Y Hirst C Stein S Walker M Cuzick J Relationship between effects on time-to-disease progression and overall survival in studies of metastatic breast cancer Br J Cancer 2008 99 1572 1578 19002178 10.1038/sj.bjc.6604759 PMC2584937

[B46] Bigelow RL Williams BJ Carroll JL Daves LK Cardelli JA TIMP-1 overexpression promotes tumorigenesis of MDA-MB-231 breast cancer cells and alters expression of a subset of cancer promoting genes in vivo distinct from those observed in vitro Breast Cancer Res Treat 2009 117 31 44 18787947 10.1007/s10549-008-0170-7

[B47] Kessenbrock K Plaks V Werb Z Matrix metalloproteinases: regulators of the tumor microenvironment Cell 2010 141 52 67 20371345 10.1016/j.cell.2010.03.015 PMC2862057

[B48] Kuvaja P Hulkkonen S Pasanen I Soini Y Lehtonen S Talvensaari-Mattila A Paakko P Kaakinen M Autio-Harmainen H Hurskainen T Lehenkari P Turpeenniemi-Hujanen T Tumor tissue inhibitor of metalloproteinases-1 (TIMP-1) in hormone-independent breast cancer might originate in stromal cells, and improves stratification of prognosis together with nodal status Exp Cell Res 2012 318 1094 1103 22465225 10.1016/j.yexcr.2012.03.009

[B49] Del Casar JM Gonzalez LO Alvarez E Junquera S Marin L Gonzalez L Bongera M Vazquez J Vizoso FJ Comparative analysis and clinical value of the expression of metalloproteases and their inhibitors by intratumor stromal fibroblasts and those at the invasive front of breast carcinomas Breast Cancer Res Treat 2009 116 39 52 19241156 10.1007/s10549-009-0351-z

[B50] Gonzalez LO Pidal I Junquera S Corte MD Vazquez J Rodriguez JC Lamelas ML Merino AM Garcia-Muniz JL Vizoso FJ Overexpression of matrix metalloproteinases and their inhibitors in mononuclear inflammatory cells in breast cancer correlates with metastasis-relapse Br J Cancer 2007 97 957 963 17848954 10.1038/sj.bjc.6603963 PMC2360420

[B51] Tvedskov TF Bartels A Jensen MB Paaschburg B Kroman N Balslev E Brunner N Evaluating TIMP-1, Ki67, and HER2 as markers for non-sentinel node metastases in breast cancer patients with micrometastases to the sentinel node APMIS 2011 119 844 852 22085360 10.1111/j.1600-0463.2011.02768.x

[B52] Nakopoulou L Giannopoulou I Lazaris AC Alexandrou P Tsirmpa I Markaki S Panayotopoulou E Keramopoulos A The favorable prognostic impact of tissue inhibitor of matrix metalloproteinases-1 protein overexpression in breast cancer cells APMIS 2003 111 1027 1036 14629269 10.1111/j.1600-0463.2003.apm1111105.x

[B53] Amir E Miller N Geddie W Freedman O Kassam F Simmons C Oldfield M Dranitsaris G Tomlinson G Laupacis A Tannock IF Clemons M Prospective study evaluating the impact of tissue confirmation of metastatic disease in patients with breast cancer J Clin Oncol 2012 30 587 592 22124102 10.1200/JCO.2010.33.5232 PMC5015424

[B54] Lindström LS Karlsson EK Wilking UM Johansson U Hartman J Lidbrink EK Hatschek T Skogg L Bergh J Clinically used breast cancer markers such as estrogen receptor, progesterone receptor, and human epidermal growth factor receptor 2 are unstable throughout tumor progression J Clin Oncol 2012 30 2601 2608 22711854 10.1200/JCO.2011.37.2482

[B55] Jensen JD Knoop A Ewertz M Laenkholm AV ER, HER2, and TOP2A expression in primary tumor, synchronous axillary nodes, and asynchronous metastases in breast cancer Breast Cancer Res Treat 2012 132 511 521 21667123 10.1007/s10549-011-1610-3

[B56] García MF Gonzalez-Reyes S González LO Berdize N Del Casar JM Medina M Vizoso FJ Comparative study of the expression of metalloproteinases and their inhibitors in different localizations within primary tumors and in metastatic lymph nodes of breast cancer Int J Exp Path 2010 91 324 334 20412339 10.1111/j.1365-2613.2010.00709.x PMC2962891

